# Experiences recruiting from neighborhoods based on area deprivation index (ADI)

**DOI:** 10.1002/alz.092673

**Published:** 2025-01-09

**Authors:** Eunji Russ, Amy J.H. Kind, Daniel L Gillen, Megan G. Witbracht, Marina Ritchie, Edwin Duran, Zion T Grant‐Freeman, Melany Medina, Joshua D Grill

**Affiliations:** ^1^ Institute for Memory Impairments and Neurological Disorders, University of California, Irvine, Irvine, CA USA; ^2^ Center for Health Disparities Research, University of Wisconsin School of Medicine and Public Health, Madison, WI USA; ^3^ University of California, Irvine, Irvine, CA USA

## Abstract

**Background:**

Few interventions have demonstrated efficacy for increasing diversity in Alzheimer’s disease (AD) clinical research and fewer still have targeted disadvantaged neighborhoods specifically. As part of a larger study to test recruitment strategies into the UC Irvine Consent‐to‐Contact Registry (C2C), we qualitatively assessed experiences of conducting community‐based outreach throughout disadvantaged neighborhoods in Orange County, California.

**Method:**

We are conducting an interrupted time series design to test recruitment interventions, stratified by Area Deprivation Index (ADI), a validated measure of neighborhood disadvantage. The experiment tests Facebook advertisements, mailed postcards, and in‐person outreach. We report the experiences of in‐person outreach in diverse neighborhoods in the first year of outreach for a two‐year study. We used the ADI to characterize the location of 223 Orange County community‐based organizations, including, 36 churches, 42 senior centers, 47 libraries, and 98 other venues. Outreach events at community‐based organizations or other venues were targeted across ADI quintiles during the first week of every study period. Our diverse team of research staff, faculty, community Speaker’s Bureau members, and undergraduate volunteers were trained to conduct outreach in the community to provide education on brain health and information about research opportunities via resource tables and educational presentations.

**Result:**

Since February 2023, we have conducted 30 events (14 presentations and 16 tables). Seven events were provided in a non‐English language (i.e., Spanish, Korean, Mandarin, and Vietnamese). We reached 205 people from tabling and 359 from presentations. Among 564 individuals reached, n = 33 provided information to register for the registry on site and n = 18 registered for a specific research study. Twenty‐two individuals reported difficulty in registering for the registry due to the absence of an email address, unfamiliarity with QR codes, or the need for family assistance to complete online forms (64% of these were from the two highest ADI quintiles). Site‐specific barriers included lack of media equipment, promotional support from site, and space for outreach.

**Conclusion:**

Identifying barriers to effective community‐based outreach in diverse neighborhoods will be important to developing strategies in improving brain health communication and research on recruitment of underrepresented communities.

